# Chemical Constituents and Antioxidant, Anti-Inflammatory and Anti-Tumor Activities of *Melilotus officinalis* (Linn.) Pall

**DOI:** 10.3390/molecules23020271

**Published:** 2018-01-29

**Authors:** Yu-Ting Liu, Pei-Han Gong, Feng-Qin Xiao, Shuai Shao, Da-Qing Zhao, Ming-Ming Yan, Xiu-Wei Yang

**Affiliations:** 1 Department of the Research and Development Center of Traditional Chinese Medicine and Biotechnology, Changchun University of Chinese Medicine, Changchun 130117, China; 18243015381@163.com (Y.-T.L.); 15543568141@163.com (P.-H.G.); 15764370851@163.com (F.-Q.X.); ss36038612@163.com (S.S.); cnzhaodaqing@126.com (D.-Q.Z.); 2 State Key Laboratory of Natural and Biomimetic Drugs (Peking University), Department of Natural Medicines, School of Pharmaceutical Sciences, Peking University Health Science Center, Peking University, Beijing 100191, China

**Keywords:** *Melilotus officinalis* (Linn.) Pall, *p*-hydroxybenzoic acid-4-*O*-α-d-manopyranosyl-(1 → 3)-α-l-rhamnopyranoside, 4-*O*-α-l-rhamnopyranosyl-(1 → 6)-α-d-manopyranosyl-(1 → 3)-α-l-rhamnopyranoside, antioxidant, anti-inflammatory, anti-tumor

## Abstract

Two new p-hydroxybenzoic acid glycosides, namely p-hydroxybenzoic acid-4-*O*-α-d-manopyranosyl-(1 → 3)-α-l-rhamnopyranoside (compound **1**) and 4-*O*-α-l-rhamnopyran-osyl-(1 → 6)-α-d-manopyranosyl-(1 → 3)-α-l-rhamnopyranoside (compound **2**), and seven known compounds, compound **3**, **6**, **7** (acid components), compound **8**, **9** (flavonoids), compound **4** (a coumarin) and compound **5** (an alkaloid), were isolated from the 70% ethanol aqueous extract of the aerial parts of *Melilotus officinalis* (Linn.) Pall. The structures of all compounds were elucidated by use of extensive spectroscopic methods Infrared Spectroscopy (IR), High resolution electrospray ionization mass spectrometry (HR-ESI-MS), and ^1^H and ^13^C-NMR). Sugar residues obtained after acid hydrolysis were identified by high-performance liquid chromatography (HPLC). The antioxidant activity of all the compounds was evaluated by 2,2′-azino-bis(3-ethylbenzothiazoline-6-sulfonic acid) (ABTS^+^) and 1,1-diphenyl-2-picrylhydrazyl (DPPH). The anti-inflammatory effects of the compounds were also evaluated in lipopolysaccharide (LPS)-stimulated RAW 264.7 macrophages. All compounds were shown to inhibit LPS-induced nitric oxide (NO) and prostaglandin E 2 (PGE 2) production by suppressing the expression of inducible NO synthase (iNOS) and cyclooxygenase-2 (COX-2), respectively, in LPS-stimulated RAW 264.7 cells. The inhibitory effect of all the compounds on MCF-7 cells was determined by Cell Counting Kit-8 (CCK-8) method. The results showed that compounds **1**, **2**, **7**, **8**, **9** exhibited better antioxidant activity compared to the other compounds. compounds **1**–**9** had different inhibitory effects on the release of NO, TNF-α and IL-6 in LPS-stimulated RAW264.7 cells by LPS, of which compound **7** was the most effective against inflammatory factors. compounds **1** and **2** have better antitumor activity compared to other compounds. Further research to elucidate the chemical composition and pharmacological effects of *Melilotus officinalis* (Linn.) Pall is of major importance towards the development and foundation of clinical application of the species.

## 1. Introduction

*Melilotus officinalis* (Linn.) Pall belongs to the genus Melilotus of Fabaceae family, and is an annual herb. It is also known as yellow sweet clover. It was first published in the “European Pharmacopoeia” eighth edition [1], widely distributed around the world. It was regarded as a drug to against edema and renal vein circulation in the UK, *Melilotus officinalis* (Linn.) Pall as a drug against aggregation, as well as for it antioxidative and hepatoprotective properties in the Netherlands, Germany, Poland and Austria [2,3,4]. In Japan, SETUS-M, which is produced with *Melilotus officinalis* (Linn.) Pall, has a good effect for treating post-surgical tissue swelling. In China, *Melilotus officinalis* (Linn.) Pall is used for the treatment of diseases such as spleen disease, gutting, diphtheria and larvae [5]. Meanwhile, the extract of *Melilotus officinalis* (Linn.) Pall achieved good results as an in-hospital preparation of Jilin University and has been widely accepted by patients.

Modern research shows that the *Melilotus officinalis* (Linn.) Pall contains coumarins [6], flavonoids [7], steroids and saponins, phenolic acids [2], volatile components, fats, alcohols, uric acid [8] and other chemical compounds, with anti-inflammatory, swelling, and anti-tumor properties, as well as with therapeutic effects against hemorrhoids, thrombophlebitis, and varicose veins [9,10,11,12]. The coumarin, phenolic acids, flavonoids and saponins of *Melilotus officinalis* (Linn.) Pall have a certain anti-inflammatory effect [8], however, these studies are mainly focused on extracts of *Melilotus officinalis* (Linn.) Pall, which involve a few monomers of the above-mentioned compounds. Therefore, 70% ethanolic extracts of *Melilotus officinalis* (Linn.) Pall were used as the research object, and the isolation, purification, identification and activity study of the monomer compounds were carried out in order to provide the basis for the clinical application.

## 2. Results and Discussion

### 2.1. Chemical Components and Monosaccharide Compositions 

#### 2.1.1. Identification of Chemical Composition

The 70% ethanol extract of yellow sweet clover was isolated by column chromatographic (CC) fractionation to give compounds **1** and **2**, together with seven known compounds: salicylic acid (compound **3**) [13], coumarin (compound **4**) [14], betaine (compound **5**) [15], fumalic acid (compound **6**) [16], and caffeic acid (compound **7**) [17]. luteolin (compound **8**), quercetin (compound **9**) [18].

**Figure 1:**
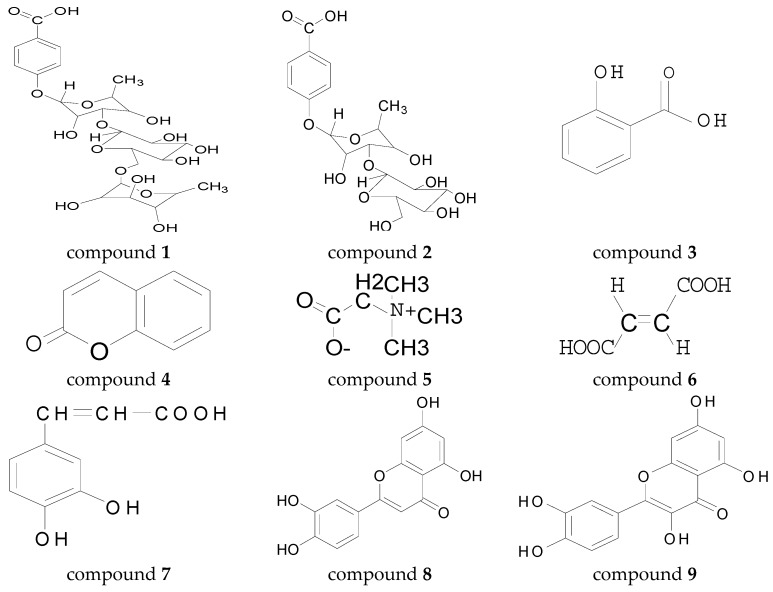
Structures of compounds **1**–**9**.

Characterizations of compound **1** included: White amorphous powder, its IR spectrum exhibited absorption bands due to -COOH at 3364, 1677 cm^−1^ and dihydrogen ortho aromatic ring at 1588, 1284, 1155, 856 cm^−1^. The HR–ESI–MS of **1** indicated the molecular formula C_19_H_26_O_12_ (*m*/*z* 469.1307 [M + Na]^+^, calcd. for C_19_H_26_O_12_Na, 469.1322). In the NMR spectra (Table 1), two proton signals at δ_H_ 8.00 (2H, d, *J* = 7.8 Hz) and 6.73 (2H, d, *J* = 7.8 Hz), and four tertiary carbon signals at δ_C_ 130.6, 130.6, 115.9, 115.9, two quaternary carbon signals at δ_C_ 121.1, 161.2, one carbonyl carbon signal at δ_C_ 175.8, combining the IR and 2D NMR spectra data, suggested that a *p*-hydroxybenzoic acid moiety existed in the structure of compound **1**. The above-mentioned ^13^C-NMR data were very similar with these of *p*-hydroxybenzoic acid reported [19], which confirmed existence of a *p*-hydroxybenzoic acid moiety in the structure of compound **1**. The ^13^C-NMR spectrum of compound **1** showed also two six-carbon units, one was characteristic of d-mannosyl group (a methylene carbon signal at δ_C_ 59.8 and five methines carbon signals at δ_C_ 102.9, 71.4, 70.5, 67.6, 73.4) which was coincident with these of methyl *O*-α-d-mannoside reported [20], and other one was characteristic of l-rhamnosyl group (a methyl carbon signal at δ_C_ 18.0 and five methines carbon signals at δ_C_ 97.8, 70.0, 75.7, 71.8, 69.7) which was coincident with these of methyl *O*-α-l-rhamnoside reported [20], except for carbon signal at δ_C_ 75.7 showing a significant downfield shift (Δδ = 4.6) than C-3 signal of α-l-rhamnose. The existence of d-mannosyl and l-rhamnosyl groups in the structure of was also confirmed by TLC comparing the acid hydrolysate of compound **1** with authentic samples. The proton signal at δ_H_ 5.41 was determined to be anomeric proton of rhamnosyl group by cross peak (Figure 1 and Table 1) at δ_H_ 5.41 (rha-H-1′)/δ_C_ 97.8 (rha-C-1′) in HMQC spectrum, and cross peak at δ_H_ 5.41/δ_C_ 161.2 (C-4) in HMBC spectrum revealed linkage of l-rhamnosyl group with 4-OH. The proton signal at δ_H_ 5.09 (Mann-H-1) was determined to be anomeric proton of d-mannosyl group by cross peak at δ_H_ 5.09/δ_C_ 102.9 (mann-C-1′′) in HMQC spectrum, and cross peak at δ_H_ 5.09/δ_C_ 75.7 (Rha-C-3′) in HMBC, revealed linkage of C-3 of l-rhamnosyl group with C-1 of d-mannosyl group, this illustrated the carbon signal at δ_C_ 75.7 (Rha-C-3′) showing a significant downfield shift (Δδ = 4.6) than C-3 signal in these of l-rhamnose reported [20]. Thus, compound **1** was determined to be *p*-hydroxybenzoic acid-4-*O*-α-d-manopyranosyl-(1 → 3)-α-l-rhamnopyranoside, a new compound.

**Figure 2:**
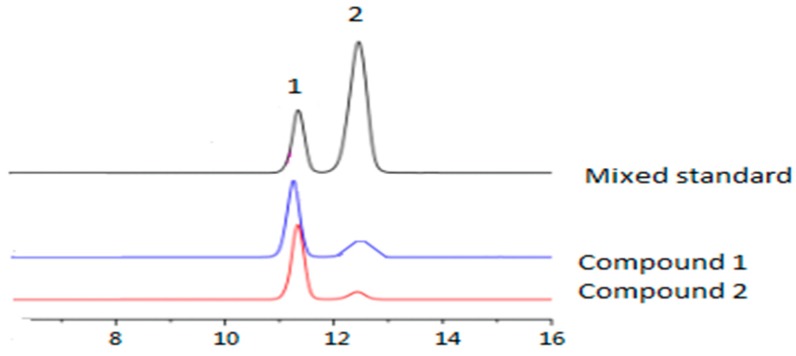
Monosaccharide analysis of compounds **1** and **2.**

Characterizations of compound **2** included: White amorphous powder. Its IR spectrum was similar with that of **1**. The molecular formula C_25_H_36_O_16_ was derived from the positive-ion mode HR-ESI-MS [M + Na]^+^ at *m*/*z* 615.1807. In the NMR spectra (Table 1) of compound **2**, the appearance of two proton signals at δ_H_ 7.97 (2H, d, *J* = 7.8 Hz) and 6.69 (2H, d, *J* = 7.8 Hz), and seven carbon signals at δ_C_ 121.1, 130.69, 130.7, 116.0, 116.0, 161.2, 175.8 showed the existence of the same aglycone (*p*-hydroxybenzoic acid) in the structure of compound **2** as in Table 1. The ^13^C-NMR spectrum of compound **2** showed three six-carbon units. Acid hydrolysis of compound **2** gave rhamnose and mannose which identified by TLC comparing with authentic samples. In comparison with the NMR data of compound **1**, two of three six-carbon units were coincident with the sugars moiety of compound **1**, except for carbon signal at δ_C_ 65.6 showing a significant downfield shift (Δδ = 5.9) than C-6 signal of d-mannosyl group of compound **1**, which point to the existence of *p*-hydroxybenzoic acid-4-*O*-*α*-d-mannopyranosyl-(1 → 3)-*α*-l-rhamnopyranosyl moiety in the structure of compound **2**, the ^13^C-NMR data (carbon signals at δ_C_ 100.1, 70.0, 70.4, 72.0, 68.2, 17.9) of the remainder six-carbon unit was coincident with these of methyl-*O*-*α*-l-rhamnoside reported [20], which point to one rhamnosyl group more than compound **1** in compound **2**, the proton signal at δ_H_ 4.40 was determined to be anomeric proton of the rhamnosyl group by cross peak (Figure 2 and Table 1) at δ_H_ 4.40 (Rha-H-1′′′)/δ 100.2 (Rha-C-1′′′) in the HMQC spectrum, the cross peak at δ_H_ 4.40/δ_C_ 65.7 (Mann-C-6′′) in the HMBC spectrum, revealed linkage of C-6 of d-mannosyl group with C-1 of l-rhamnosyl group, that illustrated the carbon signal at δ_C_ 65.7 showing a significant downfield shift (Δδ = 5.8) than C-6 signal of d-mannosyl group of compound **1**. Thus, the compound **2** was determined to be *p*-hydroxybenzoic acid-4-*O*-*α*-l-rhamnopyranosyl-(1 → 6)-*α*-d-manopyranosyl-(1 → 3) -*α*-l-rhamnopyranoside. It was a new compound.

#### 2.1.2. Monosaccharide Analysis

Compound **1** and compound **2** showed similar monosaccharide composition (Figure 2). Only two monosaccharides were found, namely d-mannopyranose and l-rhamnopyranosyl. 

### 2.2. Biological Activity 

It had been reported that benzoic acid derivatives showed antioxidant activity, anti-inflammatory [21], and cytotoxic activities [22,23]. In this work, the antioxidant activity, anti-inflammatory, and cytotoxic activities of compounds **1**–**9** was investigated. 

#### 2.2.1. Anti-Oxidative Activity

The compounds **1**–**9** was used in ATBS^+^ free radical scavenging assay. The semi-inhibitory concentration (IC_50_) of compounds **1**–**9** for ATBS^+^ and DPPH· free radical scavenging activity could be seen in Table 1.

#### 2.2.2. Anti-Inflammatory Activity

In the inflammatory response of RAW264.7 cells stimulated by LPS, compounds **1**–**9** had different inhibitory effects on the release of NO, TNF-α and IL-6 in LPS-stimulated RAW264.7 cells by LPS, and showed good anti-inflammatory activity in vitro, in which compound **7** was effective against inflammatory factors. The strongest inhibitory effect, the results shown in Table 2.

#### 2.2.3. Antitumor Activity

**Figure 3:**
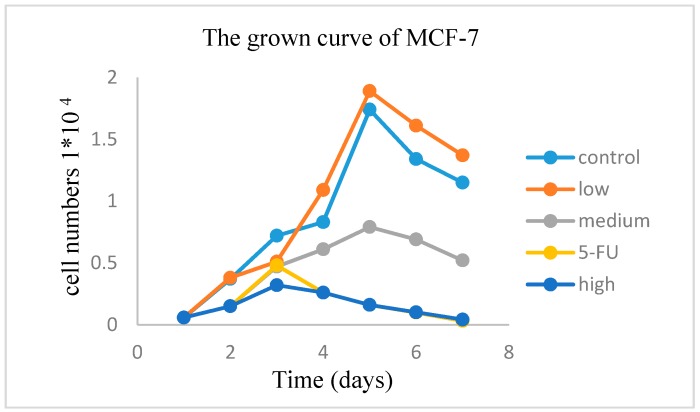
The growth curve of MCF-7 of compound **1**.

**Figure 4:**
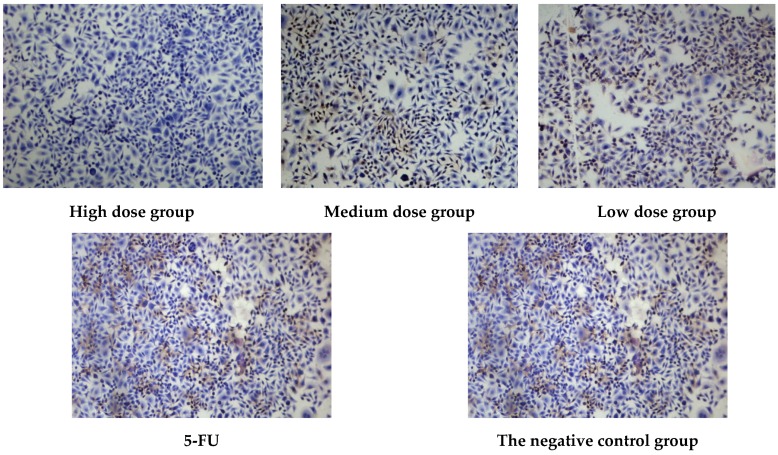
Effects of compounds on the growth of MCF-7 cells.

The results of compounds of CCK-8 kit assay are displayed in Table 3, the results show that compounds **1**, **2**, **3**, **5**, **7**, **8** and **9** can inhibit the growth of tumor cells MCF-7 with IC_50_ value of 4.83, 5.18, 8.20, 7.85, 7.53, 8.40 and 9.24 μg/mL. However, compounds **4** and **6** did not inhibited potently the growth of MCF-7 cells. According to IC_50_ values, compound **1** with the best antitumor activity was divided into three groups: low dose group (1/2 IC_50_ value), medium dose group (IC_50_ value), high dose group (2 × IC_50_ value), the concentration of 5-FU was IC_50_, which is positive control group, and the negative control group was not given the drug (0 mg·mL^−1^). Each concentration in parallel with 3 copies, 37 °C, 5% CO_2_ incubation. The number of viable cells was counted after staining with trypan blue after digestion with trypsin at the same time point. Each set of data is expressed as an average number of cells. The growth curve was plotted with the culture time as the horizontal axis and the average number of cells as the vertical axis. MCF-7 results showed that the compound **1** had a significant dose-dependent effect on the growth of MCF-7 cells, which was significantly different from that of the control group (*p* < 0.05), and there was no significant difference compared with the existing positive control drug 5-FU (*p* > 0.05) (See in Figure 3). The expression of PCNA was observed by immunohistochemical staining of MCF-7 cells; it was found that the expression of PCNA decreased gradually with the increase of concentration (See in Figure 4).

### 2.3. Discussion

Two new *p*-hydroxybenzoic acid glycosides, namely *p*-hydroxybenzoic acid-4-*O*-α-d-manopyranosyl-(1 → 3)-α-l-rhamnopyranoside (compound **1**) and 4-*O*-α-l-rhamnopyranosyl-(1 → 6)-α-d-manopyranosyl-(1 → 3)-α-l-rhamnopyranoside (compound **2**), three acid components, two flavonoids, one coumarin and one alkaloid were isolated in this study. Most of the compounds from *Melilotus officinalis* (Linn.) Pall possessed anti-oxidation, anti-tumor and anti-inflammatory effects.

*Melilotus officinalis* (Linn.) Pall has anti-inflammatory [9], swelling [10] and anti-tumor [11] and other pharmacological effects. This study showed that its flavonoids and phenolic acids have good antioxidant capacity, which suggest that the flavonoids and phenols acid composition is the material basis to antioxidant of *Melilotus officinalis* (Linn.) Pall. The phenolic acids achieved anti-inflammatory effects by inhibiting the activity of NO, TNF-α and IL-6 in LPS-induced RAW264.7 cells, which suggest that the treatment of edema with *Melilotus officinalis* (Linn.) Pall is related to its antioxidant and anti-inflammatory properties. Earlier studies have shown it has good anti-tumor activity [18]. In previous studies, our group studied the purification process of its saponins, and applied for a patent; we also found that its saponins had the better inhibitory effect on MCF-7, PC3M and other tumor cell lines. In this paper, we found two new benzoic acid compounds have good inhibitory activity on prostate cancer, and the inhibitory effect is stronger than the other compounds. In clinical applications, *Melilotus officinalis* (Linn.) Pall is mainly used to address swelling, which suggests its antitumor activity maybe has a certain correlation with therapeutic effect of its edema, and also shows that it has potential value in anti-tumor applications. Therefore, further research to elucidate the chemical composition and pharmacological effects of *Melilotus officinalis* (Linn.) Pall is of major importance towards the development and foundation of clinical application of the species.

## 3. Experimental Section

### 3.1. Materials

#### 3.1.1. Chemicals and Reagents

IR spectra were recorded using a Bruker Vertex 70 Fourier Transform Infrared Spectrometer (FT-IR) spectrometer (Bruker Company, Rheinstetten, Germany) with KBr disks. ^1^H-NMR, ^13^C-NMR, Distortionless Enhancement by Polarization Transfer (DEPT), ^1^H-^1^H Correlated Spectroscopy (^1^H-^1^H COSY), Heteronuclear Multiple Quantum Correlation (HMQC), and Heteronuclear Multiple Bond Correlation (HMBC) experiments were performed on an Bruker AVANCE 600 spectrometer (Bruker BioSpin AG, Rheinstetten, Germany; 600 MHz for ^1^H-NMR and 150 MHz for ^13^C-NMR), TMS was used as international standard, and DMSO-*d*_6_ as solvent. High-performance liquid chromatography (HPLC) was performed using an Agilent 1100 Series HPLC system (Agilent Technologies Inc., Santa Clara, CA, USA) equipped with a four-pump with an in-line degasser, autosampler, oven and Ultraviolet detector (UVD). HR-ESI-MS were measured on IonSpec 7.0 T Fourier Transform Ion cyclotron resonance mass spectrometry (FT-ICR-MS) spectrometer (Bruker Daltonics Inc., Billerica, MA, USA). Column chromatography was performed with silica gel (200–300 mesh) (Qingdao Marine Chemical Factory, Qingdao, China). Thin Layer Chromatography (TLC) was carried out with glass precoated silica gel plates (Qingdao Marine Chemical Factory, Qingdao, China). Sephadex LH-20 was used for the column chromatography (Pharmacia, 25–100 μm). D101 Macroporous resin (Tianjin Resin Technology Co., Ltd., Tianjin, China). Spots were visualised by spraying with 10% sulphuric acid in EtOH followed by heating. Solvents were analytical grade and purchased from Beijing Chemical Company, Beijing, China. standard monosaccharides (D-Gal, D-Ara, L-Fuc, L-Rha, D-Man, D-Xyl, D-Glc, D-Glc UA and D-Gal UA)were purchased from Sigma. 2-Diphenyl-1-(2,4,6-trinitrophenyl)hydrazyl (DPPH), 2-Acrylamido-2-methylpropane sulfonic acid (ATBS^+^), lipopolysaccharides (LPS) were purchased from Sigma Chemical Co. (St. Louis, MO, USA), penicillin G, streptomycin, l-glu-tamine and Dexamethasone (DEX) were purchased from local pharmaceutical industry.

#### 3.1.2. Cell-Lines

Human breast adenocarcinoma cell line MCF-7, human prostate cancer cell line PC-3M and RAW264.7 cell were obtained from Shanghai Institute of Biochemistry and Cell Biology (Shanghai, China).

#### 3.1.3. Plant Materials

The aerial part of *Melilotus officinalis* (Linn.) Pall was collected from Changbai Mountainous Nature Protection Area, in April 2014, and identified by Professor Minglu Deng, Changchun university of Chinese Medicine. A voucher specimen (140817) has been deposited in the Herbarium of Changchun university of Chinese Medicine.

### 3.2. Methods

#### 3.2.1. Extraction and Isolation

The powdered aerial part of *Melilotus officinalis* (Linn.) Pall (10 kg) was extracted three times (2 h for the first and 1 h for the second as well as third) with 70% ethanol aqueous under reflux to give an ethanolic extract (2.7 kg, yield 27.00%), which was successively partitioned with H_2_O (1.5 L) and petroleum ether, chloroform, ethyl acetate, and *n*-butanol saturated with H_2_O for the five times (each time 2 L) to obtain the petroleum ether fraction (65 g; 2.40%), chloroform fraction (176 g; 6.52%), ethyl acetate fraction (35 g; 1.29%) and *n*-butanol fraction (105 g; 3.89%). The *n*-butanol soluble fraction was chromatographed over a D101 macroporous resin column, eluted with a gradient solvent system of ethanol-H_2_O (0%, 30%, 70%, 95% ethanol solution), to yield fractions 1 (7.5 g), 2 (35.6 g), 3 (38.1 g), and 4 (15.3 g). Fraction 1 was subjected to Sephadex-LH-20 eluting with MeOH to give five crude fractions A.1-A.5, fraction A.2 was recrystallized from MeOH to yield compound **5** (32 mg); fraction 2 was subjected to Sephadex-LH-20 eluting with MeOH to give ten crude fractions B.1–B.10, the crude fraction B.3 (100 mg) which containing 1 and 2 were further purified by Preparation Thin Liquid Chromatography (PTLC) over a silica gel plate (silica G 10–40 mm, 25 × 25 cm × 1.0 mm) using CHCl_3_/MeOH/H_2_O (65:36:10) lower Placing below 10 °C as a developing system to give compound **1** (47 mg) and compound **2** (36 mg); the petroleum ether fraction heated in water bath at 90 °C to obtain compound **3** (27 mg) with sublimation method; the remaining petroleum ether fraction was chromatographed on silica gel column eluting with chloroform (CHCl_3_)/ethyl acetate (EtOAc) in gradient (10:1 to 10:5) to give four fractions C.1–C.4, fractions C.2 was further purified by PTLC over a silica gel plate (silica G 10–40 mm, 25 × 25 cm × 1.0 mm) using CHCl_3_/EtOAc/HCOOH (10:5:0.5) as a developing system to give compound **4** (35 mg); the chloroform fraction was chromatographed on silica gel column eluting with CHCl_3_/MeOH in gradient (100:0 to 80:20) to give five fractions D.1–D.5, fraction D.1 was recrystallized from MeOH to give compound **6** (12 mg), fraction D.2 was recrystallized from MeOH to obtain compound **7** (15 mg). The ethyl acetate fraction was chromatographed on silica gel column eluting with CH_2_Cl_2_/MeOH in gradient (20:1 to 0:1) to give five fractions E.1–E.5, E.3 was separated by octadecylsilyl (ODS) column chromatography and eluted with a gradient of 30% to 100% methanol to give three fractions E 3.1–E 3.3, fractions E.3.2 (500 mg) was separated by Sephadex LH-20 column and the same fractions were combined to give two fractions E.3.2.1 and E.3.2.2, fractions E.3.2.1 and E.3.2.2 were purified by semipreparative HPLC to yield compounds **8** (30 mg) and **9** (15 mg), respectively.

#### 3.2.2. *p*-Hydroxybenzoic Acid-4-*O*-α-d-manopyranosyl-(1 → 3)-α-l-rhamnopyranoside (**1**)

White amorphous powder (Methanol); m.p. 214.0–216.5 °C; IR (KBr) *ν*_max_ (cm^−1^): 3364, 1677, 1588, 1483, 1425, 1284, 1155, 1135, 1030, 856; ^1^H (DMSO-*d*_6_, 600 MHz) and ^13^C-NMR (DMSO-*d*_6_, 150 MHz) spectral data, see Table 4; HR-ESI-MS: *m*/*z* 469.1307 [M + Na]+ (calcd. for C_19_H_26_O_12_Na: 469.1322).

#### 3.2.3. *p*-Hydroxybenzoic acid-4-*O*-α-l-rhamnopyranosyl-(1 → 6)-α-d-manopyranosyl-(1 → 3)-α-l-rhamnopyranoside (**2**)

White amorphous powder(Methanol); m.p. 217.2–219.0 °C; IR (KBr) *ν*_max_ (cm^−1^): 3363, 1677, 1605, 1588, 1481, 1423, 1308, 1282, 1153, 1133, 1030, 854; ^1^H (DMSO-*d*_6_, 600 MHz) and ^13^C-NMR (DMSO-*d*_6_, 150 MHz) spectral data, see Table 4; HR–ESI–MS: *m*/*z* 615.1807 [M + Na]^+^ (calcd. for C_25_H_36_O_16_Na: 615.1823).

#### 3.2.4. Salicylic Acid 

White solid powder. The molecular formula was C_7_H_6_O_3_, m.p. 252~254 °C. ^1^H-NMRδ: 7.83 (1H, d, *J* = 8.0 Hz, H-6), 7.41 (1H, m, H-4), 6.89 (1H, d, *J* = 8.4 Hz, H-5), 6.82 (1H, m, H-3).

#### 3.2.5. Coumarin

Colorless column crystal. The molecular formula was C_9_H_6_O_2_, m.p. 68~70 °C. EI-MS *m*/*z*: 146 [M^+^], 118 [M^+^-CO], 90; ^1^H-NMR δ: 6.43 (1H, d, *J* = 9.5 Hz, H-3), 7.48 (2H, q, *J* = 8.5 Hz, *J* = 2.5 Hz, H-6, H-8), 7.52 (2H, q, *J* = 8.5 Hz, *J* = 2.5 Hz, H-5, H-7), 7.71 (1H, d, *J* = 9.5 Hz, H-4).

#### 3.2.6. Betaine

Characterizations of compound **5** included: White crystals, the formula was C_5_H_11_NO_2_, m.p. 301~305 °C. IRν^KBr^_max_ (cm^−^^1^): 3023, 2985, 1621, 1492, 1471, 1422, 1395, 1339, 1238, 1120, 982, 930, 870, 720, 603; MS *m*/*z*: 117 [M]^+^. ^1^H-NMRδ: 3.28 (9H, s, 3×-CH_3_), 3.80 (2H, s, -CH_2_); ^13^C-NMRδ: 97.0 (-N-CH_3_), 108.2 (-N-CH_2_), 210.2 (C=O).

#### 3.2.7. Fumalic Acid

Yellow block crystals, the formula was C_4_H_4_O_4_, m.p.: 296.8~299.2 °C. ^1^H-NMRδ: 13.10 (2H, s, OH-1, OH-4), 6.64 (2H, s, H-2, H-3); ^13^C-NMRδ: 166.4 (C-1), 134.4 (C-2), 134.4 (C-3), 166.4 (C-4).

#### 3.2.8. Caffeic Acid

Light yellow powder, m.p. 199.1~201.4 °C. ^1^H-NMRδ: 7.51 (1H, d, *J* = 16.0 Hz, H-7), 7.03 (1H, s, H-2), 6.92 (1H, d, *J* = 8.0 Hz, H-5), 6.72 (1H, d, *J* = 8.0 Hz, H-6), 6.22 (1H, d, *J* = 16.0 Hz, H-8). ^13^C-NMRδ: 171.6 (C-9), 149.4 (C-7), 148.6 (C-3), 145.4 (C-4), 128.3 (C-1), 126.5 (C-6), 115.1 (C-2, 5), 113.7 (C-8).

#### 3.2.9. Luteolin

Yellow needle crystal, m.p. 235~238 °C. EI-MS *m*/*z*: 286 [M^+^], 153, 134. ^1^H-NMRδ: 6.23 (1H, d, *J* = 2.1 Hz, H-6), 6.51 (1H, d, *J* = 2.1 Hz, H-8), 6.56 (1H, s, H-3), 6.97 (1H, d, *J* = 8.3 Hz, H-5′), 7.43 (1H, dd, *J* = 2.3, 8.3 Hz, H-6′), 7.46 (1H, d, *J* = 2.3 Hz, H-2′). 

#### 3.2.10. Quercetin

ESIMS (-ve) *m*/*z*: 301 [M−H]^−^; ^1^H-NMRδ:δ6.17 (1H, d, *J* = 2.0 Hz, H-6), 6.37 (1H, d, *J* = 2.0 Hz, H-8), 6.87 (1H, d, *J* = 8.0 Hz, H-5′), 7.62 (1H, dd, *J* = 2.0, 7.5 Hz, H-6), 7.73 (1H, d, *J* = 2.0 Hz, H-2′).

#### 3.2.11. Monosaccharide Analysis

A solution of each Compound (**1** or **2**) (5 mg) in a mixture of 1:2 (*v*/*v*) 1M H_2_SO_4_–MeOH (20 mL), was heated under reflux for 3 h in a water bath at 80 °C. The reaction mixture was evaporated to dryness in vacuo, dissolved in H_2_O (5 mL), and neutralized with NaOH. Then, the resulting samples were analyzed using high-performance liquid chromatography (HPLC) coupled with an ELSD detector according to the method of Yang [24], with some modifications. Instead of the gradient elution, an isocratic mobile phase consisting of 22:78 (*v*/*v*) mixtures of water and acetonitrile (ACN) was used.

#### 3.2.12. Anti-Oxidative Activity

The methods for determining ATBS^+^ free radical scavenging activity was as follows. About 0.2 mL tested compounds at various concentrations (0.01, 0.05, 0.1, 0.15, 0.20, 0.25, 0.30, 0.35, 0.40 and 0.45 mg/mL) were added to 2 mL ATBS^+^ solution, respectively. The mixture, protected from light, was reacted for 30 min. The decrease of absorbance was monitored at 734 nm. The control was 0.2 mL of distilled water and 2 mL of ATBS^+^ solution. The same method was used in Vitamin C (Vc). The methods for determining DPPH· free radical scavenging activity was as follows. About 0.2 mL tested compounds at various concentrations (0.01, 0.05, 0.1, 0.15, 0.20, 0.25, 0.30, 0.35, 0.40 and 0.45 mg/mL) were added to 2 mL DPPH· solution (200 µM ethanol solution), respectively. The mixture, protected from light, was reacted for 30 min. The decrease of absorbance was monitored at 517 nm. The control was the DPPH· solution. The same method was used in Vc. The half maximal inhibitory concentration (IC_50_) was used to evaluated the ATBS^+^ free radical scavenging activity and the DPPH· free radical scavenging activity.

#### 3.2.13. Anti-Inflammatory Activity

The rat macrophage RAW264.7 cell line was maintained in dulbecco’s modified eagle medium (DMEM) supplemented with 10% heat inactivated fatal bovine serun (FBS), penicillin G (100 units/mL), streptomycin (100 mg/mL) and l-glutamine (2 mM). The cells were grown in a humidified atmosphere containing 5% CO_2_ at 37 °C. RAW264.7 cells were seeded in 96-well plates at a density of 8 × 10^4^ cells/well for 24 h. The cells were randomly divided into control group, LPS (1 μg/mL) group, LPS (1 μg/mL) + compound **1**–**9** (50 μg/mL) group. After adding the corresponding drug, the supernatant was used to detect NO, TNF-α and IL-6 after culturing for 24 h at 5% CO_2_ and 37 °C under saturated humidity.

#### 3.2.14. Cytotoxicity Assay

The cytotoxicity assay was carried out using CCK-8 method. MCF-7 and PC-3M cells were cultured in Roswell Park Memorial Institute (RPMI) 1640 and DMEM at 37 °C in 5% CO_2_, respectively. The cells of logarithmic growth phase were seeded into 96-well plates with density of 1 × 10^4^ cells/well in 100 μL medium, respectively. The cells were treated with the tested compounds at various concentrations (0.5, 1.0, 2.5, 5, 7.5, 10.0, 12.5 and 15.0 μg/mL) and 5-FU as positive control, each of two parallel holes are located, then incubated for 72 h. Subsequently, remove the 96 well plate, add 10 μL of CCK-8; meanwhile, two separate holes for the blank control, only added to each well with 10 μL CCK-8 in DMEM 0.1 mL. Then incubate under the same conditions for 4 h. The optical density (OD) was measured at 490 nm using a Bio-red 550 (Bio-red company, Hercules, CA, USA). Reference wavelength was 620 nm. The experiment was repeated 3 times. Calculation of the impact of drugs on cell growth inhibition rate and IC_50_ values is performed with the following equation:

Growth inhibition rate (100%) = (D_0_ − D_1_)/D_0_ × 100%

where D_0_ is the OD value of the control wells, and D_1_ is the OD value of the samples wells.

## Figures and Tables

**Table 1:** The IC_50_ of compounds **1**–**9** for 2-Acrylamido-2-methylpropane sulfonic acid (ATBS^+^) and 2-Diphenyl-1-(2,4,6-trinitrophenyl)hydrazyl (DPPH) free radical scavenging activity.

	The IC_50_ of ATBS^+^ Free Radical Scavenging Activity (μg/mL)	The IC_50_ of DPPH Free Radical Scavenging Activity (μg/mL)
VC	70.00	39.06
compound **1**	25.20	53.00
compound **2**	69.75	73.00
compound **3**	166.00	143.7
compound **4**	148.00	254.1
compound **5**	87.23	176.4
compound **6**	79.83	>450
compound **7**	18.00	23.04
compound **8**	13.60	23.89
compound **9**	<10	<10

**Table 2:** Effects of compounds on production of NO, TNF-α, and IL-6 in LPS-stimulated RAW264.7 cells.

	Concentration/(μg·mL^−^^1^)	NO/(μmol·mL^−^^1^)	TNF-α/(ng·mL^−^^1^)	IL-6/(ng·mL^−^^1^)
Control		0.6045 ± 0.0098	0.0173 ± 0.0025	0.0005 ± 0.0000
LPS		6.7458 ± 0.3428 ^##^	45.3633 ± 0.2559 ^##^	0.6046 ± 0.0045 ^##^
LPS + compound **1**	50	4.0904 ± 0.4424 **	29.8130 ± 0.1658 **	0.5232 ± 0.0030 **
LPS + compound **2**	50	5.0565 ± 0.2452 **	27.5663 ± 0.1122 **	0.3047 ± 0.0040 **
LPS + compound **3**	50	0.1751 ± 0.0353 **	26.0250 ± 0.2000 **	0.2144 ± 0.0026 **
LPS + compound **4**	50	3.6667 ± 0.2301 **	30.8603 ± 0.1000 **	0.2771 ± 0.0050 **
LPS + compound **5**	50	3.0282 ± 0.4208 **	28.5656 ± 0.1000 **	0.2166 ± 0.0035 **
LPS + compound **6**	50	2.0452 ± 0.3327 **	31.8536 ± 0.1000 **	0.3136 ± 0.0025 **
LPS + compound **7**	50	0.1243 ± 0.1461 **	22.6661 ± 0.1528 **	0.2065 ± 0.0021 **
LPS + compound **8**	50	3.5865 ± 0.2452 **	28.4363 ± 0.1721 **	0.3757 ± 0.0034 **
LPS + compound **9**	50	4.2881 ± 0.2691 **	32.4133 ± 0.0577 **	0.2881 ± 0.0066 **

^##^ *p* < 0.01 vs. control group; ** *p* < 0.01 vs. model group.

**Table 3:** Anti-proliferative activities of nine monomer compounds against two tumor cells lines (IC_50_ μg/mL).

Compound	IC_50_ (μg/mL)
	MCF-7
compound **1**	4.83
compound **2**	5.18
compound **3**	8.20
compound **4**	>15
compound **5**	7.85
compound **6**	>15
compound **7**	7.53
compound **8**	8.40
compound **9**	9.24
5-FU	3.50

**Table 4:** The ^1^H (600 MHz, in DMSO-*d*_6_) and ^13^C (150 MHz, DMSO-*d*_6_) NMR data compounds **1** and **2**.

Position	1		2	
	δ_C_	δ_H_	δ_C_	δ_H_
1	121.1 (s)		121.1 (s)	
2	130.6 (d)	8.00 (d, 2H, 7.8)	130.7 (d)	7.97 (d, 2H, 7.8)
3	115.9 (d)	6.73 (d, 2H, 7.8)	116.0 (d)	6.69 (d, 2H, 7.8)
4	161.2 (d)		161.1 (d)	
5	115.9 (d)	6.73 (d, 2H, 7.8)	116.0 (d)	6.69 (d, 2H, 7.8)
6	130.6 (s)	8.00 (d, 2H, 7.8)	130.7 (s)	7.97 (d, 2H, 7.8)
Rha-1′	97.9 (d)	5.41 (brs,1H,)	97.8 (d)	5.40 (brs,1H)
2′	70.0 (d)	3.81 (brs,1H)	70.3 (d)	3.81 (brs,1H)
3′	75.7 (d)	3.15 (m,1H)	73.3 (d)	3.12 (m,1H)
4′	71.8 (d)	3.58 (m,1H)	71.8 (d)	3.50 (m,1H)
5′	69.7 (d)	3.47 (m,1H)	69.7 (d)	3.45 (m,1H)
6′	17.9 (q)	1.11(d, 3H, 6.0)	17.9 (q)	1.15 (d, 3H, 6.0)
Man-1″	102.9 (d)	5.09 (brs,1H)	103.7 (d)	4.99 (brs,1H)
2″	71.4 (d)	3.57 (m,1H)	70.6 (d)	3.58 (m,1H)
3″	70.5 (d)	3.62 (m,1H)	71.2 (d)	3.61 (m,1H)
4″	67.6 (d)	3.69 (brs,1H)	68.1 (d)	3.63 (brs,1H)
5″	73.4 (d)	3.27 (m,1H)	73.5 (d)	3.13 (m,1H)
6″-A	59.8 (t)	3.16 (m,1H)	65.7 (t)	3.20 (m,1H)
6″-B		3.47 (brs,1H)		3.57 (m,1H)
Rha-1″′			100.2 (d)	4.40 (brs,1H)
2″′			70.0 (d)	3.80 (brs,1H)
3″′			70.4 (d)	3.43 (m,1H)
4″′			72.0 (d)	3.49 (m,1H)
5″′			68.2 (d)	3.39 (m,1H)
6″′			17.9 (q)	1.08 (d, 3H, 6.0)

All the signals were assigned by 1D and 2D NMR spectra.
